# Histone variant H2A.J is an epigenetic regulator of metastasis in lung adenocarcinoma

**DOI:** 10.1038/s41419-026-09054-9

**Published:** 2026-06-26

**Authors:** Dong-Gun Kim, Eun-Young Choi, Hye-Mi Ahn, Kyungho Kim, Youn-Jae Kim

**Affiliations:** Targeted Therapy Branch, Division of Rare and Refractory Cancer, Research Institute, National Cancer Center, Goyang, Republic of Korea

**Keywords:** Non-small-cell lung cancer, Cancer epigenetics

## Abstract

Metastasis is a major contributor to poor patient survival in lung adenocarcinoma (LUAD); however, the underlying mechanisms remain incompletely understood. Unlike tumorigenesis-associated mutations, recurrent genetic alterations specifically linked to metastasis have not been identified, suggesting that epigenetic mechanisms may play a key role. In this study, we report that histone H2A variant H2A.J expression is significantly down-regulated in LUAD, and that low H2A.J levels are associated with unfavorable survival outcomes. Functional assays revealed that H2A.J overexpression suppresses cancer cell invasion and metastatic potential by modulating the expression of metastasis-associated genes, including TMEM158. Mechanistically, H2A.J is deposited in the promoter region of TMEM158, where it alters the local chromatin status to suppress transcriptional activity. Taken together, our findings suggest that H2A.J functions as an epigenetic suppressor of metastasis in LUAD and highlights its potential as both a prognostic biomarker and a therapeutic target to metastatic progression.

## Introduction

Lung cancers are generally divided into two primary groups: non-small cell lung cancer (NSCLC) and small-cell lung cancer (SCLC). More than 85% of lung cancers are represented by NSCLC, which is further broken down into lung adenocarcinoma, squamous cell carcinoma, and large cell carcinoma. Lung adenocarcinoma (LUAD) accounts for approximately 80% of all NSCLC cases [1]. NSCLC, including LUAD, is currently the leading cause of cancer-related deaths worldwide [2].

Metastasis in LUAD occurs in approximately 50% of cases at diagnosis and is associated with high mortality rates [3, 4]. Hence, understanding the molecular mechanisms driving metastasis and identifying specific biomarkers are crucial for improving the diagnosis, treatment, and outcomes of patients with LUAD. However, no recurrent genetic mutations directly associated with these aspects of LUAD have been previously reported. This suggests that the underlying mechanisms are governed by epigenetic gene regulation [5–8]. Therefore, it is important to elucidate the epigenetic mechanisms of metastasis in LUAD to inform the development of novel therapeutic options.

Histone variants confer different structural properties on chromatin by modulating DNA accessibility and altering nucleosome stability. They exhibit specialized functions such as DNA repair, chromosome segregation, and regulation of transcription initiation as well as tissue-specific roles [9, 10]. In particular, H2A and H3 influence the transcriptional activation or repression of metastasis-related gene expression [11–13]. The histone H2A variant H2A.Z is reported to be associated with cancer cell proliferation and metastasis in breast cancer, prostate cancer, and melanoma. Another H2A variant, macroH2A, represses melanoma metastasis via direct transcriptional regulation of cyclin-dependent kinase 8 (*CDK8*) [14–18]. Additionally, histone H3 variant H3.3 promotes lung cancer cell migration by regulating the expression of G protein-coupled receptor 87 (*GPR87*) [19]. H2A.J is a DNA replication-independent H2A histone variant that participates in responses to inflammation and senescence [20, 21]. It exhibits altered expression in various malignancies and may be associated with chemotherapy resistance [22–26], however the functional role of H2A.J in LUAD metastasis remains unexplored.

In this study, we investigated *H2AJ* expression and its prognostic significance in patients with LUAD. In addition, the role of H2A.J and its regulatory function in gene expression were examined in LUAD cells. Collectively, our findings demonstrated that H2A.J plays a significant role in LUAD metastasis.

## Materials and methods

### Public microarray data

Microarray data (GSE50081, GSE31210, and GSE10072) were downloaded from the NCBI GEO database (https://www.ncbi.nlm.nih.gov/geo/). Statistical and survival analyses were performed using R software (https://www.r-progect.org). Graphs and heat maps were prepared using Microsoft Excel, R, and deepTools [27].

### Cell culture

A549 red firefly luciferase (FLuc) cells were purchased from Perkin Elmer (MA, USA) and maintained in RPMI-1640 medium supplemented with 10% fetal bovine serum (FBS) and 1% antibiotics (penicillin and streptomycin). A427 cells were purchased from Korean Cell Line Bank (Seoul, South Korea) and were maintained in RPMI-1640. HEK293 and 293 T cells were purchased from ATCC (VA, USA) and were maintained in Dulbecco’s modified Eagle’s medium (DMEM) medium supplemented with 10% FBS and 1% antibiotics. All cells were maintained at 37 °C in a humidified atmosphere of 5% CO_2_. All medium and FBS were purchased from Cytiva (MA, USA), and antibiotics were purchased from Welgene (Gyongsan-si, South Korea).

### Public Methylation data analysis

TCGA LUAD DNA methylation profiles were analyzed using the Wanderer platform (http://maplab.imppc.org/wanderer/index.html).

### Methylation bisulfite amplicon sequencing analysis

For the methylation assay, A549 and A427 cells were treated with 1 μM 5-aza-2’-deoxycytidine (5-Aza; Sigma-Aldrich, MO, USA) for 24 h. Following the treatment, genomic DNA (gDNA) was extracted using QIAamp DNA Mini Kit (Qiagen, Germany) according to the manufacturer’s instructions. The isolated gDNA was then utilized for library construction to perform methylation bisulfite amplicon sequencing.

Potentially existing sequencing adapters and raw quality bases in the low reads were trimmed using Skewer ver. 0.2.2 [28]. The cleaned high-quality reads after trimming the low-quality bases and sequencing adapters were mapped to the reference genome using BS-seeker2 ver 2.1.2 software [29]. To determine the methylation levels from the mapping results, BS-seeker2 was used. Methylation levels were calculated at single-base resolution. To compare the methylation profiles of the CpG sites among the groups of samples, the values of the two target CpG sites were selected.

### Public ChromHMM and Histone ChIP-Seq data

Public A549 chromHMM data were obtained from The NIH Roadmap Epigenomics Mapping Consortium [30]. Public A549 wild-type cell histone chromatin immunoprecipitation (ChIP)-Seq data were downloaded from the ENCODE database (https://www.encodeproject.org/; H3K4me1: ENCSR000AUM, H3K4me2: ENCSR000AVI, H3K4me3: ENCSR000ASH, H3K9ac: ENCSR000ASV, H3K9me3: ENCSR000AVN, H3K27ac: ENCSR000AUI, H3K27me3: ENCSR000AUK, H3K36me3: ENCSR000AUL). ChromHMM and ChIP-Seq results overlapping with H2A.J were calculated using bedtools [31]. ChIP-seq data were extracted to a bigwig file and visualized using Integrative Genomic Viewer (IGV) [32].

### Lentiviral overexpression and shRNA knockdown

*H2AJ* CDS with an N-terminal FLAG epitope was inserted into the pHRST-IRES-GFP vector. Myc-DDK-tagged TMEM158 was purchased from Origene (MD, USA) and was inserted into the pLenti-V6 vector. Lentiviruses were generated from cloned lentiviral plasmids by co-transfecting 293 T cells with packaging vectors VSV-G and delta8.9. For overexpression, 1.5 × 10^5^ lung cancer cells were seeded into six-well plates, incubated overnight, and infected with lentivirus. After 24 h of viral infection, the medium was replaced with a complete medium and incubated for 48 h. Three days after viral infection, western blotting and another assay were performed.

The siRNA target sequences were designed using BLOCK-iT™ RNAi Designer, and the sequences of the transfected siRNAs are listed in Suppl. Table S1. For siRNA transfection, 1.5 × 10^5^ lung cancer cells were seeded into six-well plates and incubated overnight. siRNAs and non-targeting controls were transfected into lung cancer cells by using Lipofectamine 2000 in Opti-MEM. After 4 h of incubation, the medium was replaced with a complete medium and incubated for 48 h. After 2 days of siRNA transfection, proliferation and invasion assays and western blotting were performed.

### RNA extraction and quantitative real-time PCR (qRT-PCR) analysis

Total RNA was isolated using the RNeasy Mini Kit (QIAGEN, Hilden, Germany) according to the manufacturer’s protocol. Reverse transcription was performed using 1 μg of total RNA as a template with SuperScript III Reverse Transcriptase (Invitrogen, MA, USA). qRT-PCR was performed in triplicate on a LightCycler 480 (Roche, Basel, Switzerland) using the LightCycler 480 SYBR Green I Master Mix (Roche, Basel, Switzerland). Gene expression levels were normalized to those of *gapdh*, and the values represent the mean ± standard error of the mean (SEM) from independent experiments. Primers were designed manually or using the Primer3 program (https://primer3.ut.ee), and are listed in Supplementary Table S2.

### Immunoprecipitation (IP) and immunoblot analysis

Whole cell lysates were prepared using RIPA buffer (iNtRON Biotechnology, Seongnam-si, South Korea) with a protease inhibitor cocktail (Roche, Basel, Switzerland). Total protein was quantified using the Pierce BCA Protein Assay Kit (Thermo Fisher Scientific, MA, USA). For IP, the cell lysates were incubated with FLAG antibody overnight at 4 °C and subsequently incubated with protein G Sepharose for 3 h at 4 °C with gentle rocking. The samples were centrifuged and washed thrice with lysis buffer. The samples were then added to sample buffer and boiled for 5 min [33]. For immunoblotting, equal amounts of protein lysates were separated on 8–16% Bis-Tris protein gels (Invitrogen, MA, USA), transferred to PVDF membranes (Millipore, Darmstadt, Germany), and blocked with 5% skim milk (BD Biosciences, NJ, USA). The primary antibodies used are listed in Supplementary Table S3. HRP-conjugated anti-mouse and -rabbit IgG antibodies (Bio-Rad, CA, USA) were used as the secondary antibody. Specific bands were detected using the WEST-ZOL plus Western Blot Detection System (iNtRON Biotechnology, Seongnam-si, South Korea).

### Invasion assay

Following procedures previously described in [19], Transwell chambers (Corning, NY, USA) were coated with Matrigel Basement Membrane Matrix (BD Biosciences, NJ, USA). Cells were suspended in serum-free medium and seeded into the upper chamber at a density of 2.0–5.0 × 10^4^ cells per well, and serum-containing medium was placed into the lower chamber. After 24 h of incubation, the cells penetrating the pores were stained with Diff-Quick staining solution (Sysmex, Kobe, Japan). Three random fields were photographed in each chamber, and stained cells were counted in the field.

### Proliferation assay

The cells were plated in 96-well plates at a density of 2 × 10^3^ cells/well. Following incubation for 0–72 h, a combination of CyQUANT NF Cell Proliferation dye reagent and deliverer (Invitrogen, MA, USA) was introduced and incubated at 37 °C for 30 min [33]. Fluorescence intensity was quantified as the fluorescence ratio at 530 nm to that at 485 nm using an infinite M200 Pro microplate reader (TECAN, Zurich, Switzerland).

### In vivo tumor models

Following the procedures previously described in [33], this animal study protocol was approved by the Institutional Animal Care and Use Committee of the National Cancer Center (Approval # NCC-20-423) and conducted in accordance with the ARRIVE guidelines [34]. Ten nude mice (BALB/c-nude, 5-week-old females) were purchased from Orient Bio (Seongnam-si, South Korea). After one week, mice were randomly divided into Control and H2AJ groups, 1.0 × 10^6^ vehicle or *H2AJ*-overexpressed lung cancer cells resuspended in 100 μL of phosphate-buffered saline (PBS) were intravenously administered via tail vein injection. Tumor metastasis was monitored by weekly bioluminescence imaging using IVIS Lumina III (PerkinElmer, MA, USA). Mice were euthanized 18 days after cancer cell injection, and lung metastasis from the transplanted tumor was observed under a microscope. The number of metastatic nodules in the transplanted tumors in the lungs was counted. Lung tissues were prepared for hematoxylin and eosin (H&E) staining to assess transplanted tumor metastasis.

### RNA-Seq analysis

Sequencing libraries for RNA-Seq were prepared from 2 μg of total RNA using a TruSeq Stranded mRNA Sample Preparation Kit (Illumina, CA, USA), according to the manufacturer’s protocol. Paired-end 101 bp reads were sequenced using the HiSeq 2500 Sequencing System (Illumina, CA, USA). RNA-Seq data were analyzed using the HISAT2 and stringTie [35, 36] programs for short-read gapped alignment to the GRCh37/hg19 reference genome. Differential gene expression analysis was performed using DESeq2 [37] based on these count values, and Gene Set Enrichment Analysis (GSEA) was performed with the hallmark (H), curated (C2), and ontology (C5) gene sets obtained from MSigDB [38]. Signal values were generated from the alignment of reads (BAM file) using deepTools [27] and visualized using IGV.

### Chromatin immunoprecipitation assay

Following the procedures previously described [19], ChIP was performed using the EZ ChIP Assay Kit (Millipore, Darmstadt, Germany) according to the manufacturer’s instructions. For ChIP-PCR/qPCR, 1.0–4.0 × 10^6^ cells were cross-linked with 1% formaldehyde for 10 min at 25 °C. For ChIP-Seq, 1.0 × 10^7^ cells were prepared for cross-linking and cells were lysed for 10 min at 4 °C with the SDS Lysis Buffer provided in the kit. Sonication using a focused ultrasonicator (Covaris, MA, USA) yielded 150–500 bp fragments. Samples were 10-fold diluted (1.0–4.0 × 10^6^ cells per tube) in the ChIP dilution buffer provided in the kit and pre-cleared with 60 μL of Protein G Agarose beads for 60 min at 4 °C. Primary antibodies were added to the pre-cleared supernatant, the mixture was incubated overnight at 4 °C. Sixty microliters of Protein G Agarose beads was added to the samples, and the mixtures were incubated for 1 h at 4 °C. The beads were washed with various buffers (low salt, high salt, LiCl, and TE). After 15 min of incubation at room temperature (RT), the precipitated chromatin was eluted twice in 100 μL of elution buffer (0.1 M NaHCO_3_ and 1% SDS). Reverse crosslinking was performed for 4 h at 65 °C. Thereafter, chromatin was treated with RNase A for 30 min at 37 °C and with Proteinase K for 1 h at 45 °C, fragments were purified using the kit’s column. ChIP-PCR assays were performed using the AmfiXp and PCR Master Mix, and ChIP-qPCR reactions were performed in triplicate on a LightCycler 480 machine using the LightCycler 480 SYBR Green I Master Mix. The data were normalized to the input levels.

### ChIP-Seq analysis

For ChIP-Seq analysis, sequencing libraries were generated from 1 ng of DNA using the TruSeq DNA Sample Prep Kit (Illumina, CA, USA), following the manufacturer’s instructions. Paired-end 101 bp reads were obtained using a NovaSeq 6000 Sequencing System (Illumina, CA, USA). ChIP-Seq data were analyzed using Bowtie2 [39] for mapping to the GRCh37/hg19 reference genome, and significant peaks were identified using MACS2 [40] programs for peak detection. To determine the ChIP-Seq read distribution, peak profile maps were calculated using ngsplot [41] and deepTools over a 3 kb window around the TSS and H2A.J summits from mapping the output files. Signal values were log_2_ normalized to total read counts compared to input signals, and peaks were visualized using the Integrative Genomics Viewer (IGV) and UCSC genome browser [19].

### Reporter assay

*TMEM158* (−2724 to −2129) promoter sequences were cloned into the pGL3-Basic Luciferase Reporter Vector. Following the procedures previously described [19], a luciferase assay was conducted using the Dual-Luciferase Reporter assay system (Promega, WI, USA) according to the manufacturer’s instructions. For transfection, 5.0 × 10^4^ cells were plated in each well of a 24-well plate. Luciferase reporters were co-transfected with a control plasmid encoding Renilla luciferase into plated cells. After 2 days of transfection, luciferase activity was detected. The luciferase activity was quantified using a VICTOR Light luminometer (PerkinElmer, MA, USA). Transfection efficiency was normalized to Renilla luciferase activity.

### Statistical analysis

Statistical analyses were performed using Student’s *t*-test and log-rank test, where *P* < 0.05 was considered statistically significant. Data are presented as the mean ± standard error of the mean (SEM) from at least three independent biological replicates. For survival analysis, patients were stratified into high- and low-expression groups based on a statistically optimized cutoff that yields the most significant *P*-value.

## Results

### *H2AJ* is downregulated in LUAD patients

**Fig. 1:**
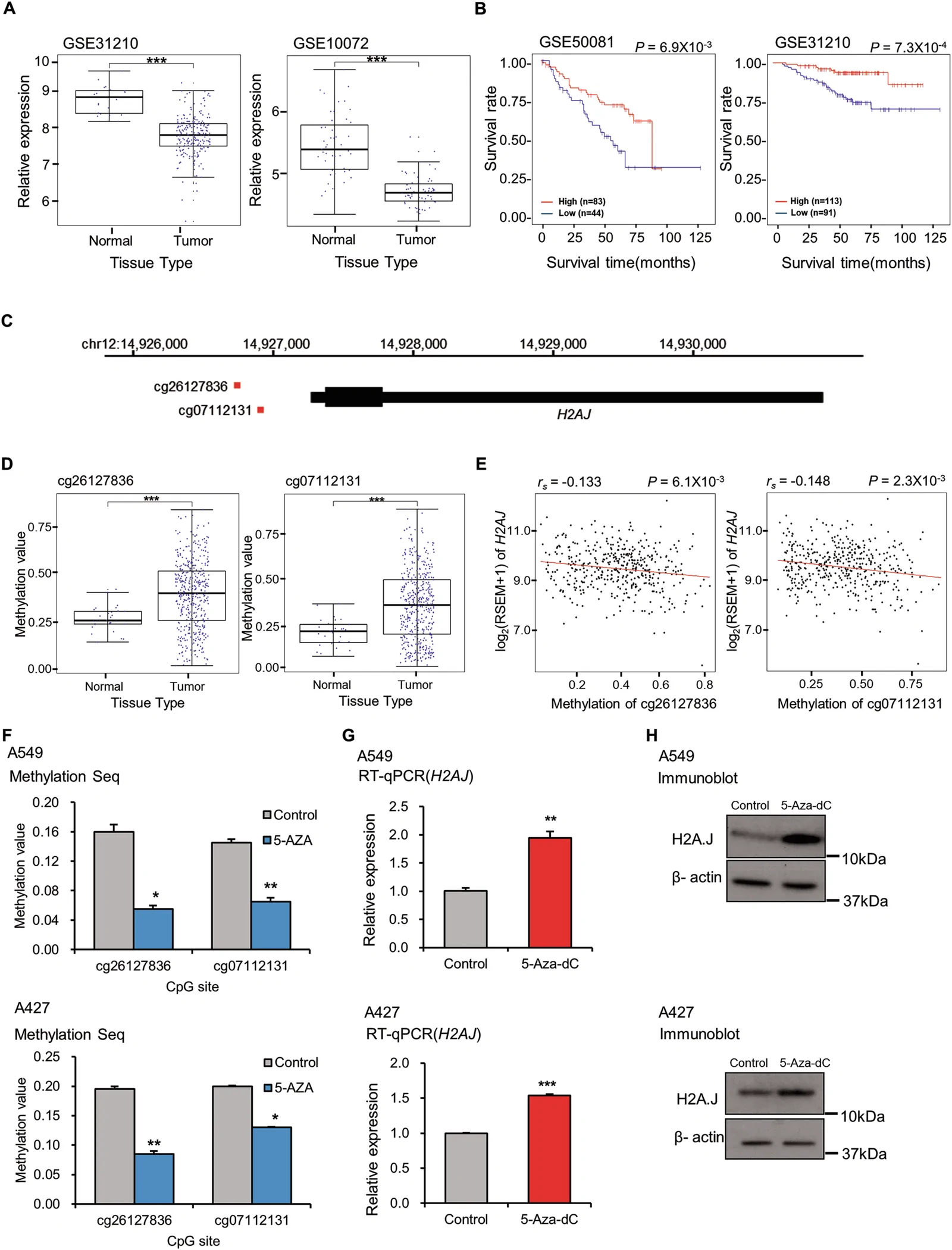
*H2AJ* expression is downregulated in LUAD. **A** Comparison of *H2AJ* expression in normal and tumor tissues of patients with LUAD (*P* = 1.2 × 10^−10^ for GSE31210 and 1.4 × 10^−14^ for GSE10072, *t*-test). **B** Overall survival rates of patients with LUAD classified by *H2AJ* expression (*P* = 6.9 × 10^−3^ for GSE50081 (*n* = 127) and 7.3 × 10^−4^ for GSE31210 (*n* = 204), log-rank test). **C** Schematic diagram of the two methylation sites in *H2AJ* promoter. **D** Comparison of DNA methylation patterns in normal and tumor tissues of patients with LUAD (*P* = 1.6 × 10^−5^ for cg26127836 and 2.4 × 10^−5^ for cg07112131, *t*-test). **E** Correlation between DNA methylation patterns and *H2AJ* expression in patients with LUAD (*P* = 6.1 × 10^−3^ for cg26127836 and 2.3 × 10^−3^ for cg07112131, Spearman’s rank correlation coefficient). **F** DNA methylation values of two methylated probes in A549 and A427 cells treated with 5-aza-deoxycytidine (5-Aza-dC) for 24 h. **G** *H2AJ* mRNA expression in A549 and A427 cells treated with 5-Aza-dC for 24 h. *H2AJ* mRNA expression levels were normalized to those of GAPDH. **H** Immunoblot validation of H2A.J in A549 and A427 cells treated with 5-Aza-dC for 24 h. Error bars show the mean ± standard error of the mean (SEM) of three biological replicates; **P < 0.01, ***P < 0.001 vs. control.

The LUAD datasets from the NCBI Gene Expression Omnibus (GEO) database revealed significantly lower *H2AJ* gene expression in tumor tissues than in normal tissues (*P* = 4.4 × 10^−11^ for GSE31210, 1.4 × 10^−14^ for GSE10072, and 5.7 × 10^−13^ for GSE19188, *t*-test, Fig. 1A, and Suppl. Fig. 1A). Using survival data to investigate the correlation between survival outcomes and *H2AJ* expression in GEO datasets revealed that low *H2AJ* expression was associated with poorer overall survival rates (*P* = 6.9 × 10^−3^ for GSE50081 (*n* = 127); *P* = 7.3 × 10^−4^ for GSE31210 (*n* = 226); *P* = 1.4 × 10^−2^ for GSE19188 (*n* = 40), log-rank test, Fig. 1B, and Suppl. Fig. 1B). These findings suggest that *H2AJ* downregulation may serve as a valuable prognostic marker for predicting unfavorable survival outcomes in patients with LUAD.

DNA methylation is an important epigenetic modification in gene regulation, development, genomic imprinting, and chromatin structure [42] associated with the silencing of tumor suppressor genes in various cancers. [43] Therefore, the Cancer Genome Atlas data were employed to examine the relationship between *H2AJ* DNA methylation and LUAD progression. Two significantly higher methylation positions were detected in tumor tissues (*P* = 1.6 × 10^−5^ for cg26127836 and *P* = 2.4 × 10^−5^ for cg07112131 (*n* = 468), *t*-test, Fig. 1C, D, and Supplementary Fig. 1C). Moreover, a weak negative correlation was identified between *H2AJ* expression and *H2AJ* DNA methylation in both datasets (*r*_*s*_ = −0.133, *P* = 6.1 × 10^−3^ for cg26127836; *r*_*s*_ = −0.148, *P* = 2.3 × 10^−3^ for cg07112131, Spearman’s rank correlation coefficient, Fig. 1E, and Suppl. Fig. 1D). To determine whether DNA methylation was responsible for the downregulation *of H2AJ* expression, we treated A549 and A427 LUAD cells with the DNA methyltransferase inhibitor 5-aza-deoxycytidine (5-Aza-dC) in vitro. This treatment lowered methylation levels at these positions and increased *H2AJ* mRNA and protein expression, underscoring the role of DNA methylation in suppressing *H2AJ* expression in LUAD (Fig. 1F–H).

### *H2AJ* regulates malignant properties of LUAD cells

**Fig. 2:**
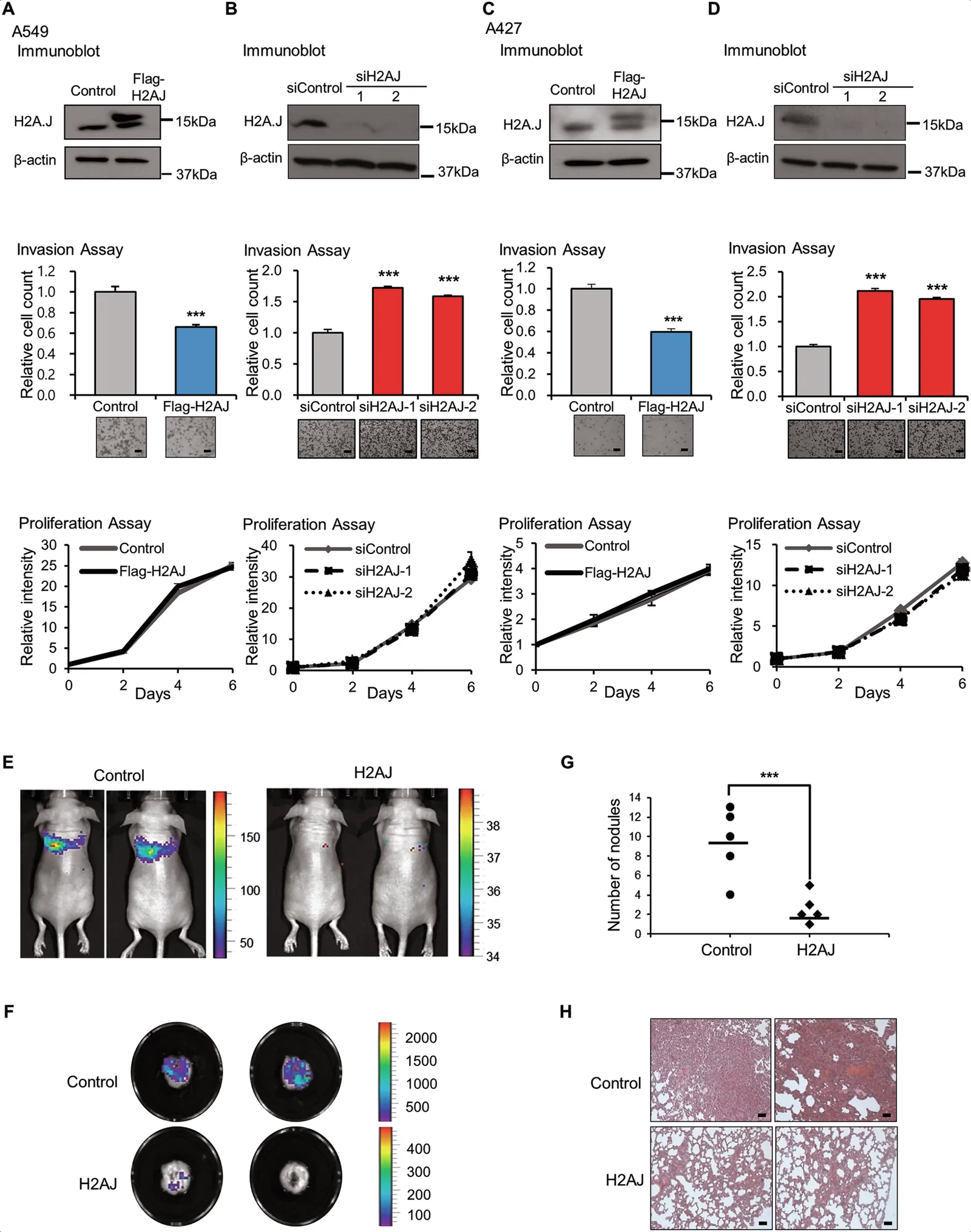
Modulation of *H2AJ* regulates malignant properties of LUAD cells. **A**, **B** Results of invasion and proliferation assays after ectopic *H2AJ* overexpression (**A**) and knockdown (**B**) in A549 cells. **C**, **D** Invasion and proliferation assay results following ectopic *H2AJ* overexpression (**C**) and knockdown (**D**) in A427 cells. **E** Luminescence signals of A549 cells in lungs of mice following intravenous injection (5 mice each group). **F** Luminescence signals of A549 cells in lungs of mice after sacrifice. **G** Metastatic nodules in lungs of mice after sacrifice (*P* = 2.2 × 10^−3^). **H** Hematoxylin and eosin (H&E) staining of lung tissues. **P < 0.01, ***P < 0.001 vs control. Scale bar = 200 μm.

We investigated tumor growth and metastatic phenotypes following modulation of *H2AJ* expression. Specifically, we assessed the changes in proliferation and invasion after *H2AJ* overexpression or knockdown in two LUAD cell lines, A549 and A427. *H2AJ* overexpression significantly suppressed cell invasion without affecting cell proliferation (Fig. 2A, C). Conversely, *H2AJ* knockdown increased cell invasion, leaving proliferation unaffected (Fig. 2B, D). *H2AJ*-overexpressing A549-Red-FLuc cells were intravenously administered into the tail veins of the mice, resulting in significantly reduced lung metastasis (Fig. 2E, F and Suppl. Fig. 2). Moreover, the number of metastatic nodules decreased with *H2AJ* overexpression (Fig. 2G, and Suppl. Fig. 3A). H&E staining of lung tissues (Fig. 2H, and Supplementary Fig. 3B) showed a decrease in the metastatic tumor region in the mouse lungs due to *H2AJ* overexpression. These results suggest that *H2AJ* expression regulates the metastatic potential without affecting growth.

**Fig. 3:**
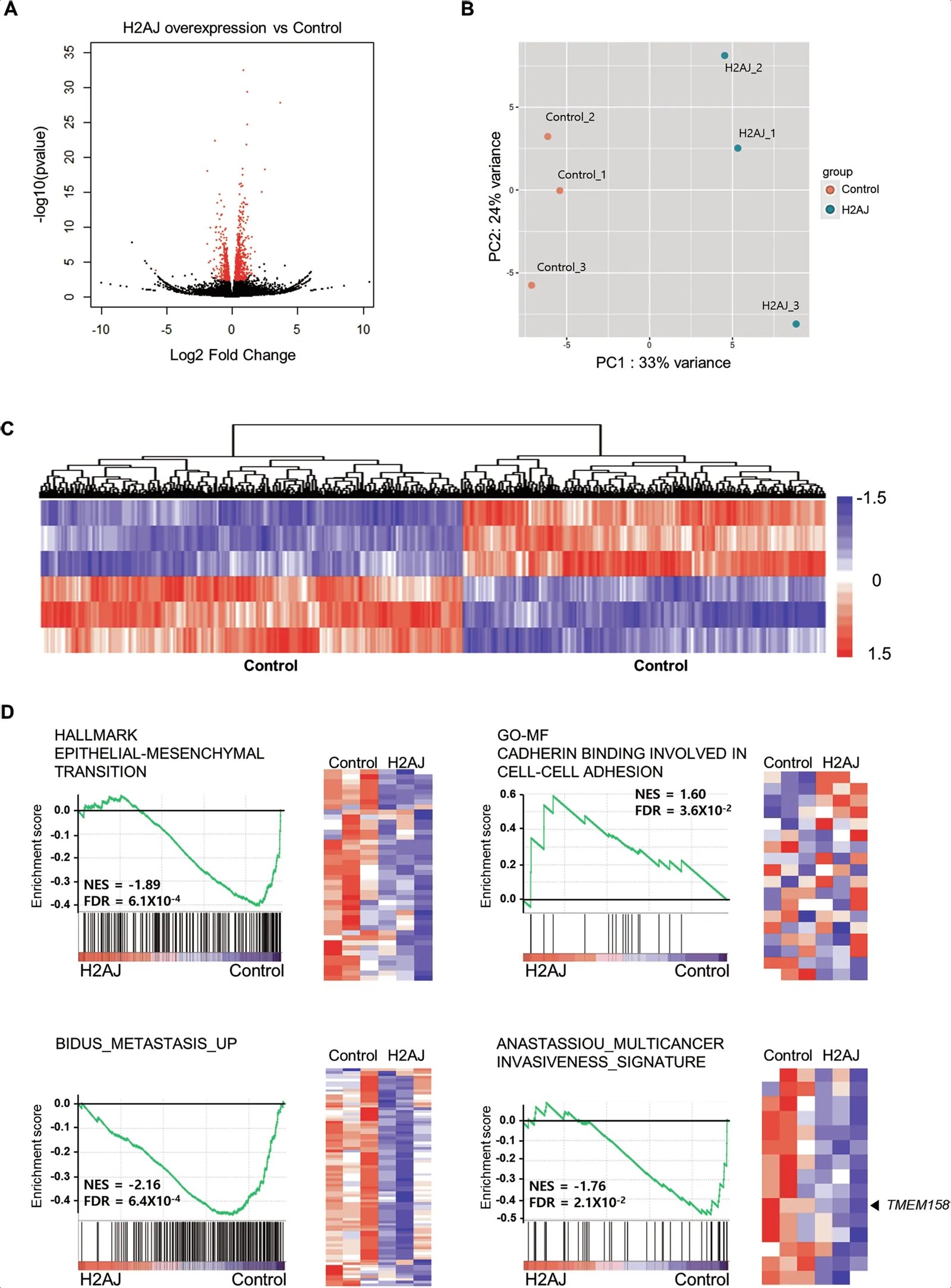
RNA-seq analysis for transcriptional regulation via H2A.J. **A**, **B** Volcano plot (**A**) and PCA plot (**B**) of RNA-seq data following ectopic *H2AJ* overexpression in A549 cells. **C** Heatmap of the DEGs (497 genes are upregulated, 428 genes are downregulated, *FDR* < 0.05). **D** Gene set enrichment analysis (GSEA) of RNA-seq data following ectopic *H2AJ* overexpression in A549 cells.

Considering that *H2AJ* encodes the histone H2A variant H2A.J, which regulates the transcription of downstream target genes [21], we hypothesized that H2A.J regulates the transcription of various invasion-related genes in LUAD. We performed RNA-Sequencing (RNA-Seq) to test this hypothesis using *H2AJ*-overexpressing A549 cells. *H2AJ*-overexpressing cells showed a change in the expression of 497 upregulated genes and 428 downregulated genes compared to control cells (Fig. 3A–C, Suppl. Table 4). We performed Gene Set Enrichment Analysis (GSEA) to compare the altered gene expression and enhanced metastatic signatures of LUAD due to *H2AJ* overexpression. Notably, the expression of gene sets related to epithelial-to-mesenchymal transition and metastasis signatures was decreased by *H2AJ* overexpression in LUAD cells, whereas the expression of the cadherin-involved cell-cell adhesion gene set was increased (Fig. 3D), suggesting that H2A.J acts as a regulator of the metastatic process by regulating the expression of associated genes.

### H2A.J is related to repressive epigenetic marks

**Fig. 4:**
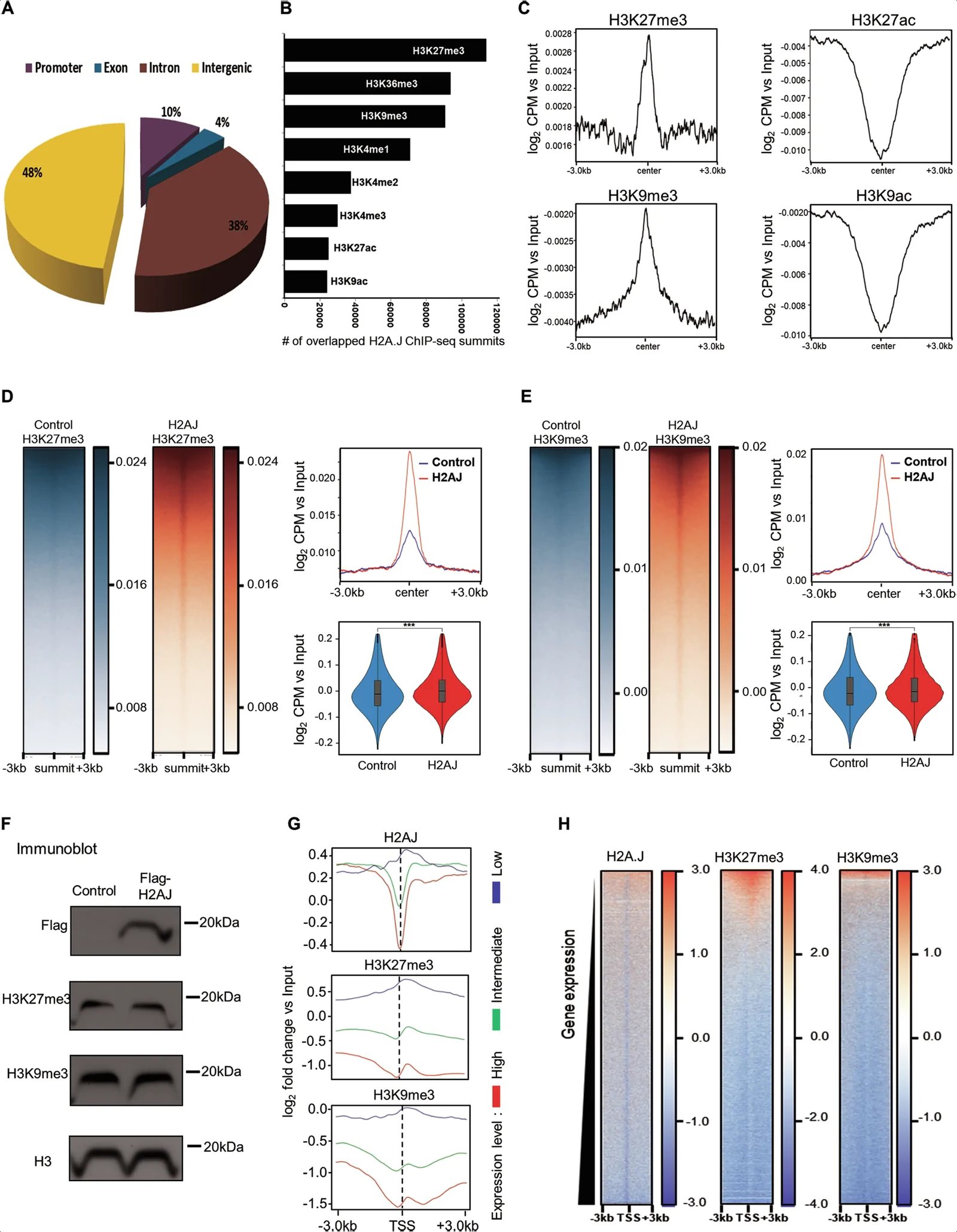
H2A.J is associated with repressive histone modifications. **A** Genomic distribution of anti-Flag ChIP-Seq peaks in *H2AJ* overexpressing A549 cells. Promoter indicates 3 kb regions upstream and downstream of the TSSs. Intergenic refers to regions larger than 3 kb from TSS or TTS. **B** Association of H2A.J peaks with various histone markers. Data are shown as the number of H2A.J binding summits overlapping the peak regions of these histone marks. **C** ENCODE histone ChIP-Seq signal profiles in the H2A.J binding summit region. **D**, **E** Heatmaps and violin plots of H3K27me3 (**D**) and H3K9me3 (**E**) ChIP-Seq signal profiles at the H2A.J binding summit region in control or *H2AJ* overexpressing A549 cells. **F** Immunoblot validation of Flag-H2A.J, H3K27me3 and H3K9me3 after ectopic *Flag*-*H2AJ* overexpression in A549 cells. **G**, **H** Flag-H2A.J, H3K27me3, and H3K9me3 ChIP-Seq read distribution profiles (**G**) and heatmaps (**H**) of three gene groups classified by their expression levels (red: high-expression group, green: intermediate-expression group, and blue: low-expression group, *n* = 5565, 5567, and 3260 genes).

We reasoned that H2A.J might function as a regulator of gene expression similar to other histone variants. Thus, to characterize the genome-wide occupancy of H2A.J in LUAD cells, we conducted a chromatin immunoprecipitation (ChIP)-seq assay in *H2AJ*-overexpressing A549 cells using both FLAG and H2A.J antibodies. After analyzing peak locations in the human genome using input DNA as an internal control, we confirmed that the two ChIP-seq peaks were highly overlapped (Suppl. Fig. 4A, B). The human genome comprises approximately 20,000 coding genes (https://grch37.ensembl.org/Homo_sapiens/Info/Annotation), and their promoter regions ( ± 3 kb from the transcription start site (TSS)) occupy ~4% of the total human genome (Suppl. Fig. 4C) [44]. However, 10% of the H2A.J peaks were localized to the promoter region of the A549 genome (Fig. 4A, and Suppl. Fig. 4D). These results suggest that elevated promoter binding of H2A.J contributes to the regulation of target gene expression.

The occupancy of certain histone variants at promoters influences histone modifications [45, 46]. Thus, to identify the overlap between H2A.J and histone modifications, we acquired histone ChIP-Seq data from the Encyclopedia of DNA Elements (ENCODE) database (https://genome.ucsc.edu/ENCODE/). A strong overlap was detected for H2A.J with histone modifications such as tri-methylation of histone H3 lysine 27 (H3K27me3) and tri-methylation of histone H3 lysine 9 (H3K9me3), which are typical repressive histone marks. In contrast, a weak overlap was observed for typical active histone modifications, such as the acetylation of histone H3 lysine 27 (H3K27ac) and acetylation of histone H3 lysine 9 (H3K9ac) (Fig. 4B, and Suppl. Fig. 4E). In addition, we combined A549 chromHMM analysis data with H2A.J ChIP-seq data to determine which regions of H2A.J occupancy were predominantly represented. Similar to the above results, our results revealed that the genomic occupancy of H2A.J predominantly occurs in quiescent states (such as intergenic and intronic regions) but is also abundant in genomic regions where transcription is repressed (polycomb and weak transcription status). (Supplementary Fig. 4F). To verify the association between H2A.J and H3K27me3 or H3K9me3, we examined a ChIP-seq assay using anit-H3K9me3 or -H3K27me3 antibodies and analyzed the histone modification ChIP-Seq profiles near the H2A.J summits. H2A.J peaks were positively correlated with repressive histone markers and negatively correlated with the active marker peaks (Fig. 4C, and Supplementary Fig. 4G). In addition, the overlap between H2A.J and the repressive markers H3K27me3 and H3K9me3 was higher when *H2AJ* overexpressed (Fig. 4D, E). However, *H2AJ* overexpression did not affect global levels of these repressive histone modifications (Fig. 4F). These results suggest that changes in histone modifications caused by *H2AJ* overexpression may be selectively localized.

We then analyzed the correlation between gene expression and H2A.J occupancy. Genes were grouped into low-, intermediate-, and high-expression groups based on expression tertiles under *H2AJ* overexpression, and the ChIP-seq profiles of H2A.J, H3K27me3, and H3K9me3 were subsequently analyzed across ±3 kb regions centered on the Transcription Start Site (TSS) for each group. A negative correlation was observed between H2A.J, H3K27me3, and H3K9me3 deposition and gene expression, and these findings suggest that H2A.J may be associated with repression of gene expression, similar to other repressive histone modifications (Fig. 4G, H, and Suppl. Fig. 4H).

### H2A.J represses the transcription of *TMEM158* in LUAD cells

**Fig. 5:**
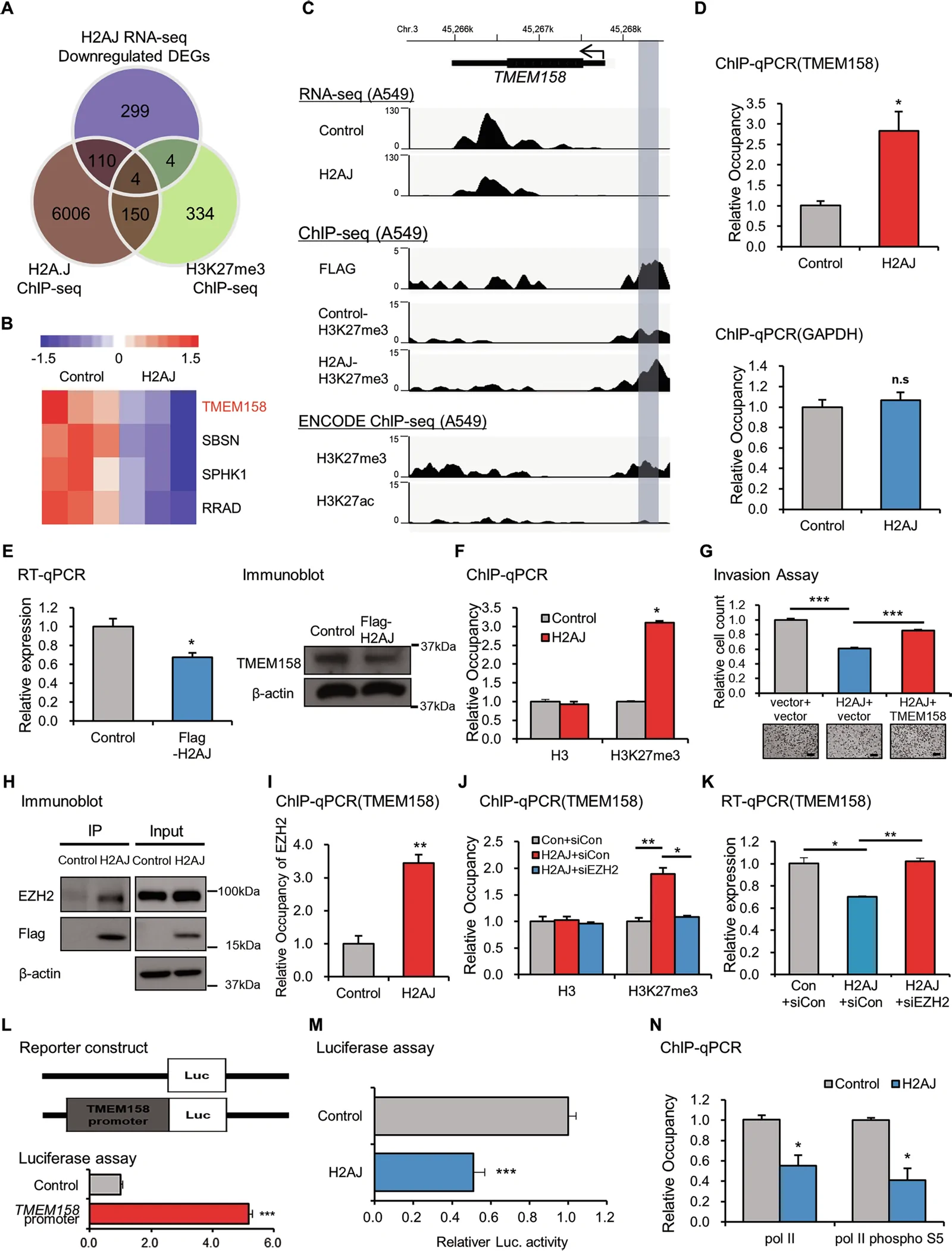
*TMEM158* is a target gene of H2A.J. **A** Results of Venn analysis between ChIP-Seq and RNA-Seq data using *H2AJ* overexpressing A549 cells. **B** Heat map of putative target gene expression changes. **C** Integrated analysis of *TMEM158* gene. The shaded blue boxes represent the promoter sites to which H2A.J binds in the ChIP-seq analysis. **D** Validation of H2A.J binding to promoter regions of *TMEM158* and unchanged gene (*GAPDH*) using a ChIP-PCR assay. **E** RT-qPCR and immunoblot validation of TMEM158 in *H2AJ* overexpressing A549 cells. **F** H3K27me3 at *TMEM158* promoter in *H2AJ* overexpression condition. **G** Results of Invasion assays with *H2AJ*, *TMEM158* overexpression alone or in combination. **H** The immunoprecipitation analysis for interaction between H2A.J and EZH2 after ectopic *H2AJ* overexpression in A549 cell. **I** Binding of EZH2 at *TMEM158* promoter in *H2AJ* overexpression condition. **J**, **K** H3K27me3 at *TMEM158* promoter (**J**) and RT-qPCR validation of *TMEM158* expression (**K**) in *H2AJ* overexpression alone or with *EZH2* knockdown. **L**, **M** Reporter construct with *TMEM158* promoter (**L**) and luciferase assay results using the reporter system with promoters in HEK293 cells (**M**). **N** Repression of transcriptional machinery on *TMEM158* promoter by *H2AJ* overexpression. Error bars show the mean ± standard error of the mean (SEM) of 3 biological replicates, *P < 0.05, **P < 0.01, ***P < 0.001 vs control; ns not significant.

Based on these results, we hypothesized that H2A.J decreases gene expression by increasing repressive histone modifications at the promoters. First, by performing a Venn diagram analysis of the ChIP-Seq and RNA-Seq data, we identified four genes that exhibited both H2A.J and H3K27me3 modifications in their promoter regions ( ± 1 kb of the TSS) and were downregulated due to *H2AJ* overexpression (Fig. 5A, B and Suppl. Fig. 5). Among these, we selected *TMEM158* for further mechanistic studies, as it is involved in cancer metastasis [47–51] and is part of the downregulated metastasis-related gene set (Fig. 3D; ANASTASIOUS MULTICANCER INVASIVENESS SIGNATURE) identified through our GSEA analysis. We found that H2A.J specifically binds to the promoter region of *TMEM158* (Fig. 5C, D, and Suppl. Fig. 5). We also examined the deposition of H2A.J in other genes and no changes in H2A.J binding were identified on the promoters of genes whose expression was not regulated by *H2AJ* overexpression (Fig. 5D, Suppl. Fig. 6A). Downregulation of *TMEM158* through *H2AJ* overexpression was validated by reverse transcription quantitative PCR (RT-qPCR) and immunoblotting in *H2AJ*-overexpressing A549 and A427 cells (Fig. 5E, Suppl. Fig. 6B). We found that H3K27me3 modification was increased on the *TMEM158* promoter in *H2AJ*-overexpressing A549 and A427 cells (Fig. 5F, Suppl. Fig. 6C). Moreover, suppression of cell invasion induced by *H2AJ* overexpression was restored by *TMEM158* overexpression and overexpression of *TMEM158* did not affect the level of Flag-H2A.J (Fig. 5G, Suppl. Fig. 6D, E). These results suggested that *TMEM158* is a functional target gene of H2A.J.

Polycomb repressive complex 2 (PRC2) mediates this methylation of H3K27me3 through the histone methyltransferase enhancer of zeste homolog 2 (EZH2) [52]. Our findings revealed that H2A.J bound to EZH2 (Fig. 5H, Suppl. Fig. 6F). Additionally, we observed enhanced EZH2 binding to the *TMEM158* promoter in *H2AJ*-overexpressing A549 cells (Fig. 5I, Suppl. Fig. 6G). To confirm the functional interaction between H2A.J and EZH2, we examined the regulation of H3K27me3 on the *TMEM158* promoter by *EZH2* knockdown under *H2AJ-*overexpression conditions. H3K27me3 accumulation on the *TMEM158* promoter induced by *H2AJ* overexpression was not observed in *EZH2* knockdown cells (Fig. 5J, Suppl. Fig. 6H), and downregulation of *TMEM158* expression by *H2AJ* overexpression was restored by *EZH2* knockdown (Fig. 5K, Suppl. Fig. 6I).

Next, to verify the regulatory role of H2A.J in gene expression, we generated reporter constructs containing *TMEM158* promoter regions, which exhibited promoter activity (Fig. 5L). The promoter activity of *TMEM158* was repressed by *H2AJ* overexpression, indicating that *H2AJ* overexpression regulates *TMEM158* transcription through interaction with its promoter region (Fig. 5M). We then conducted ChIP-qPCR analysis of RNA polymerase II (Pol II) binding to the *TMEM158* promoter to investigate whether *H2AJ* overexpression affects the transcriptional machinery of *TMEM158*. *H2AJ* overexpression significantly repressed the occupancy of Pol II and phosphorylated (phosphor S5) Pol II within its promoter region (Fig. 5N, Suppl. Fig. 6J). Taken together, these results suggest that H2A.J promotes the modification of H3K27me3 on the promoter by EZH2 binding, leading to repressed gene expression.

H2A.J’s amino acid sequence is very similar to that of canonical H2A, but H2A.J has an A11V substitution and the C-terminal motif (Suppl. Fig. 7A). Previous study shows that the C-terminal motif of H2A.J controls downstream gene expression by regulating its transcriptional activity [21]. To assess the biological function of the specific H2A.J C-terminal motif in LUAD cells, we generated an H2A.J S123A mutant (H2A.J-SA). We first investigated whether this mutation affected the H2A.J–EZH2 association at the TMEM158 promoter via overexpression of either H2A.J-WT or the mutant. Given that H2A.J represses target genes through the H2A.J–EZH2 axis, we performed ChIP-qPCR to examine EZH2 recruitment to the TMEM158 promoter. Unlike H2A.J-WT, the H2A.J-SA mutant failed to increase EZH2 binding or H3K27me3 enrichment (Suppl. Fig. 7B). Consequently, TMEM158 expression was not repressed in H2A.J-SA-expressing cells (Suppl. Fig. 7C, D), and promoter activity was significantly enhanced compared to H2A.J-WT (Suppl. Fig. 7E).

We further explored the phenotypic impact of this mutation on cell growth and invasion. Notably, H2A.J-SA did not lead to the suppression of cell invasion observed in H2A.J-WT cells, while neither construct significantly altered cancer cell proliferation (Supplementary Fig. 7F, G). Collectively, these findings suggest that the unique C-terminal motif of H2A.J is critical for its regulatory function and its ability to interact with epigenetic repressors.

### TMEM158 participates in LUAD progression

**Fig. 6:**
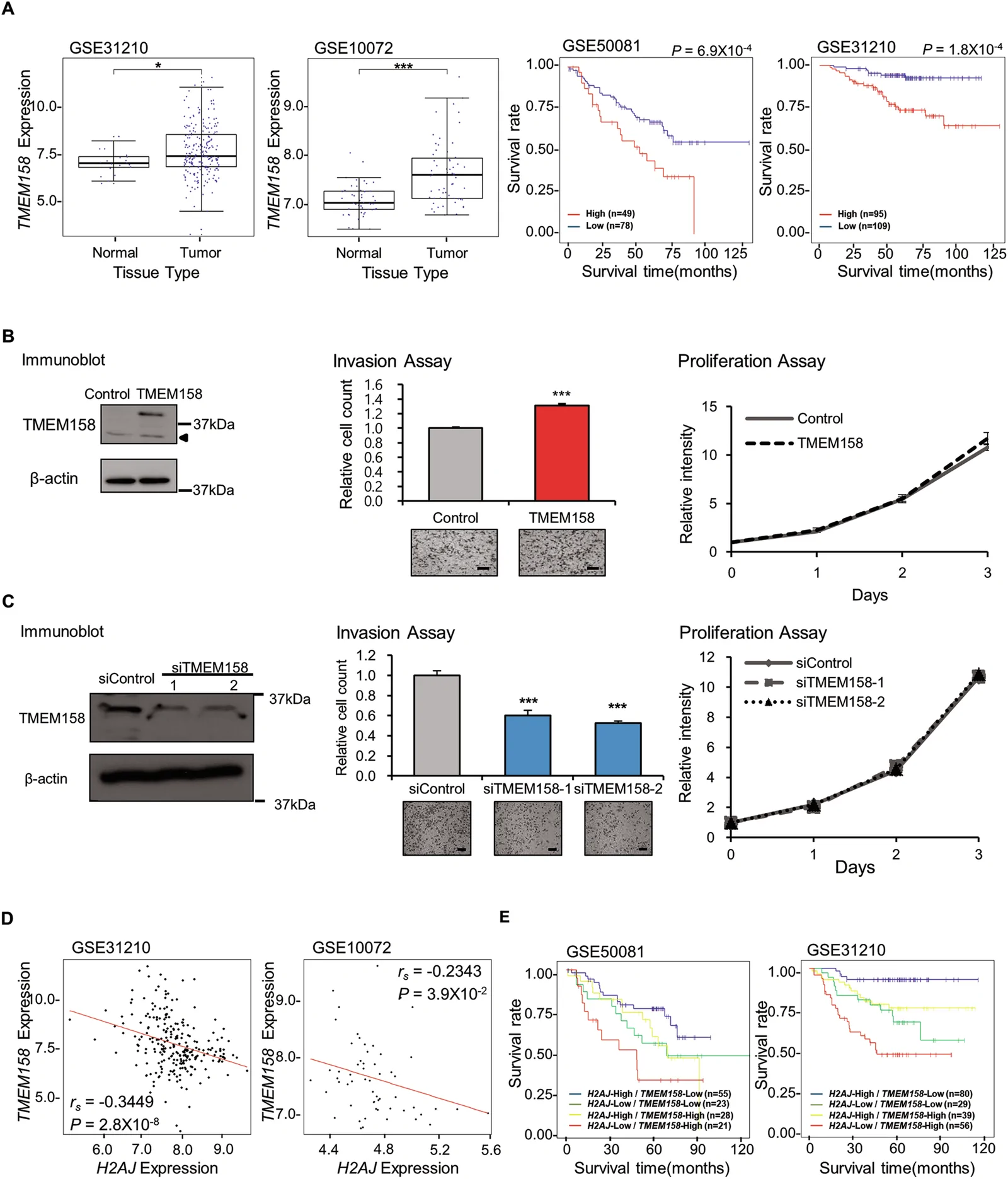
TMEM158 is associated with LUAD malignant properties. **A** Comparison of *TMEM158* expression in normal and tumor tissues of patients with LUAD (*P* = 2.7 × 10^−2^ for GSE31210 and 6.3 × 10^−7^ for GSE10072, *t*-test) and overall survival rates of these patients classified by *TMEM158* expression (*P* = 1.8 × 10^−4^ for GSE31210 and 6.9 × 10^−4^ for GSE50081, log-rank test). **B**, **C** Results of immunoblot validation, invasion, and proliferation assay after ectopic *TMEM158* overexpression (**B**) and knockdown (**C**) in A549 cells. Upper band is Flag-tagged TMEM158 proteins and lower band is endogenous proteins (black triangle). Scale bar = 200 μm. **D** Correlation of *H2AJ* and *TMEM158* expression (*P* = 2.8 × 10^−8^ for GSE31210 and 3.9 × 10^−2^ for GSE10072, Spearman’s rank correlation coefficient). **E** Overall survival of LUAD patient groups stratified by *H2AJ* and *TMEM158*. The number of patients (n) in each subgroup (*H2AJ*-high/*TMEM158*-low, *H2AJ*-low/*TMEM158*-low, *H2AJ*-high/*TMEM158*-high, and *H2AJ*-low/*TMEM158*-high) is indicated for the GSE50081 (*n* = 55, 23, 28, 21) and GSE31210 (*n* = 80, 29, 39, 56), respectively. Error bars show the mean ± standard error of the mean (SEM) of 3 biological replicates, *P < 0.05, ***P < 0.001 vs control.

To determine the clinical relevance of *TMEM158* expression in patients with LUAD, we examined the oncogenic features of *TMEM158* in previously used GEO datasets. *TMEM158* was highly expressed in tumor tissues and was associated with poor prognosis (Fig. 6A, Suppl. Fig. 8A). To estimate the biological functions of *TMEM158* in LUAD cells, we examined metastatic phenotypes following the overexpression and depletion of *TMEM158* expression in LUAD cells. *TMEM158* overexpression increased cell invasion, and loss of *TMEM158* significantly suppressed invasion without affecting cell proliferation (Fig. 6B, C, and Suppl. Fig. 8B, C). To determine the association between *H2AJ* and *TMEM158* expression and cancer progression in patients with LUAD, the datasets presented in Fig. 6A was analyzed. A significant negative correlation was observed between *H2AJ* and *TMEM158* expression (*r*_*s*_ = −0.3449, *P* = 2.8 × 10^−8^ for GSE31210; *r*_*s*_ = −0.2343, *P* = 3.9 × 10^−2^ for GSE10072, Spearman’s rank correlation coefficient) in patients with LUAD (Fig. 6D, Suppl. Fig. 8D).

We then divided the patients into four groups according to their *H2AJ*, *TMEM158* expression levels and performed overall survival analysis. Patients were stratified into four groups based on the expression levels of these genes (*H2AJ*-High / *TMEM158*-Low, *H2AJ*-Low / *TMEM158*-Low, *H2AJ*-High / *TMEM158*-High, and *H2AJ*-Low / *TMEM158*-High). For each gene, the cutoff value distinguishing high and low expression was determined by identifying the point that yielded the most statistically significant separation between the two conditions. Using these optimized cutoff points, patients were assigned to one of the four expression-based subgroups. All patients in the low *H2AJ* and high *TMEM158* expression groups had the worst prognoses. In contrast, patients in the high *H2AJ* and low target expression groups exhibited the best prognosis (Fig. 6E, Suppl. Fig. 8E). Taken together, these results indicate that H2A.J-induced regulation of *TMEM158* expression is critical for LUAD progression.

## Discussion

Previous research indicates the absence of recurrent genetic mutations associated with metastasis, suggesting that LUAD metastasis may be regulated by epigenetic regulation. This study shows that the histone variant H2A.J epigenetically modulates LUAD metastasis by regulating repressive histone modifications and the subsequent repression of metastasis-related gene transcription.

H2A.J promotes immune system activation by binding to the promoter regions of inflammatory genes and those associated with the senescence-associated secretory phenotype (SASP), contributing to chronic inflammation related to aging in human fibroblast cells [21]. Additionally, accumulation of H2A.J in senescent human fibroblasts diminishes H1 expression and its association with chromatin. This leads to increased gene expression by enhancing H3K4me3 and RNA polymerase II binding in regions of interferon-stimulated genes [53]. Moreover, *H2AJ* overexpression promotes the expression of genes associated with epithelial–mesenchymal transition (EMT), TNF-α/NF-κB, and IL-6/STAT3 pathways in glioblastoma [26]. *H2AJ* expression is also positively correlated with Extracellular signal-Regulated Kinase 5 (ERK5) expression and Human immunodeficiency virus (HIV) Nef pathway markers in colorectal cancer [23]. While these previous studies indicated that *H2AJ* promotes the expression of target genes, we found that *H2AJ* overexpression downregulated the expression of metastasis-related genes and upregulated the expression of cell-cell adhesion genes in LUAD. These gene expression regulatory functions of H2A.J indicate that its properties are context- and cancer-type-dependent. Both up- and downregulation of these genes may contribute to the more complex phenotypes driven by high *H2AJ* expression in lung adenocarcinoma cells. In particular, upregulation of cell-cell adhesion gene sets by *H2AJ* overexpression suggests that the H2A.J mechanism may inhibit cancer metastasis.

A previous study based on fibroblasts showed no significant peak in the deposition of H2A.J. [21] However, our results indicate that H2A.J is localized to the promoters of repressed genes, which has not been observed in previous fibroblast-based studies. This suggests that the unique repressive function of H2A.J in LUAD may be attributed to its specific position. The difference in distribution could be caused by variations in *H2AJ* expression between different cell types or by the existence of other co-factors, and this may be worthy of further investigation.

H2A.J is a regulatory H2A histone variant that is similar to H2A.Z and macroH2A. However, it also has several unique characteristics. H2A.Z has a low amino acid sequence identity with canonical H2A (~60%); macroH2A also has an N-terminal H2A-domain ( ~ 64%) and features a unique domain sequence called the “macro domain” at the C-terminus, which reportedly indicates functional differences [54]. In contrast, H2A.J has an amino acid sequence with high similarity to canonical H2A, differing only in the A11V substitution and SQ motif near the C-terminus [21]. H2A.Z is enriched at active enhancers and around the promoter of active genes near the TSS, increasing gene expression by interacting with the transcription factor E2F, BET protein family member BRD2, or active histone variant H3.3 [17, 55]. Meanwhile, macroH2A exhibits gene silencing through strong enrichment at repressed gene loci such as silenced gene bodies, inactive enhancers, and promoters [56]. Moreover, H2A.Z exhibits the same distribution of histone modifications associated with active gene transcription, such as H3K27ac, H3K4me2, H3K4me3, and H4ac [57, 58], while macroH2A is associated with repressive histone modifications H3K9me3 and H3K27me3 [59, 60]. Similarly, our results suggest that H2A.J is enriched in the promoter region near the TSS and represses target gene expression through its association with H3K27me3.

Histone variants are involved in the regulation of several types of cancer. H2A.Z is upregulated in various cancers, including melanoma, breast, bladder, prostate, and liver cancers, and its high expression is associated with EMT regulatory proteins, transcriptional regulators, and estrogen receptor-alpha (ERα) target genes [14, 17, 56, 61]. In contrast, macroH2A is downregulated in melanoma, colon, breast, bladder, prostate, ovarian, and cervical cancers and can act as a tumor suppressor by modulating enhancer activity, repressing transcription initiation, and modifying chromatin structure [56, 62–64]. Moreover, macroH2A overexpression limits the growth of metastatic tumors in head and neck squamous cell carcinoma (HNSCC), whereas reducing macroH2A levels in melanoma cells leads to increased proliferation and metastasis [18, 65]. In lung cancer, macroH2A reduces cell proliferation by inhibiting PARP-1 expression [66], which is associated with recurrence in patient tissues [67]. Furthermore, H3.3 exhibits oncogenic characteristics that promote the expression of metastasis-related genes, such as GPR87, in LUAD [19]. However, the mechanism by which H2A.Z and macroH2A regulate metastasis has not yet been characterized, and no other histone variant that performs a metastasis suppressor function has been detected in LUAD cells. We discovered that H2A.J reduced LUAD metastasis by inhibiting the transcription of genes associated with metastasis, such as *TMEM158*. *TMEM158* is a gene known to be involved in cancer metastasis, and our results suggest that H2A.J regulates the expression of the *TMEM158* gene at an upstream level and that H2A.J and EZH2 interact to cause H3K27me3 modification at the *TMEM158* promoter. This functional occupancy of H2A.J and H3K27me3 is a gene-specific phenomenon in LUAD, but follow-up studies are needed to determine whether this function of H2A.J also applies to other cancer types and whether other genes regulated by H2A.J are involved in LUAD invasiveness.

Specialized motifs in histone variants are known to play pivotal roles in diverse nuclear processes, including DNA repair, transcriptional regulation, and cell division. For instance, phosphorylation of the H2A.X (S139) is a well-characterized marker of the DNA damage response [68]. Recent studies have similarly highlighted that mutations in the H2A.J SQ motif (S123) can significantly alter the transcriptional landscape and expression patterns of its target genes [21, 53]. Consistent with these reports, our findings suggest that the H2A.J SQ motif is essential for the regulation of downstream targets. Notably, mutation of this motif was associated with significantly reduced recruitment of the EZH2 and H3K27me3 modification. While other histone variants, such as H2A.Z and macroH2A, are known to modulate PRC2 activity by altering chromatin accessibility and H3K27me3 deposition [60, 69], our data provide evidence for a motif-specific role of H2A.J in this process. However, it should be noted that we did not directly verify the phosphorylation status of the H2A.J SQ motif in the context of LUAD, nor did we identify the precise amino acid residues that facilitate the direct physical interaction with EZH2. Nevertheless, our data provide compelling evidence that this motif is intimately linked to the EZH2-H3K27me3 regulatory mechanism, as its mutation significantly disrupted the recruitment of the PRC2 complex and subsequent histone methylation at target promoters. Beyond phosphorylation, the SQ motif may possess unique structural properties or uncharacterized functions in coordinating the assembly of epigenetic modifiers. Further investigation is required to fully elucidate the exact molecular interface and signaling pathways that govern the interaction between H2A.J and the EZH2-mediated silencing machinery.

Our analysis demonstrated that H2A.J negatively regulates the metastasis-related gene *TMEM158*. TMEM158 promotes invasion and metastasis of cancer cells. However, its potential role in regulating LUAD metastasis remains unknown. We found that *TMEM158* expression negatively affected the outcomes of patients with LUAD and cell invasion. Moreover, a combination of high *H2AJ* and low *TMEM158* expression was associated with improved LUAD prognosis, suggesting that the expression level of *TMEM158* could be used as a prognostic marker for LUAD. Enhancing *H2AJ* expression and reducing *TMEM158* expression could be strategies for decreasing LUAD-related deaths.

## Supplementary information

Suppl. Figs.

Original blot figures

Supplementary Table 1

Supplementary Table 2

Supplementary Table 3

Supplementary Table 4

## Data Availability

The RNA-Seq, ChIP-Seq, and methylation amplicon sequencing datasets generated during the current study have been deposited in the NCBI’s Gene Expression Omnibus (GEO) database under the GEO accession number GSE331098.
