# Supplementary material for: Histone variant H2A.J is an epigenetic regulator of metastasis in lung adenocarcinoma
Source: Cell Death Dis. 2026 Jun 26;17(1):791. doi: 10.1038/s41419-026-09054-9 (PMC13558708; doi:10.1038/s41419-026-09054-9)
Supplement: Supplementary file 1 [file 41419_2026_9054_MOESM1_ESM.docx]

**Histone variant H2A.J is an epigenetic regulator of metastasis in lung adenocarcinoma**

Dong-Gun Kim †, Eun-Young Choi †, Hye-Mi Ahn , Kyungho Kim, Youn-Jae Kim*

**Supplementary Fig. S1**. Validation of H2AJ expression in another LUAD dataset. **A** Comparison of H2AJ expression in normal and tumor tissues of patients with LUAD. **B** Overall survival rates of patients with LUAD classified by H2AJ expression (*P* = 1.4 × 10^-2^ for GSE19188 (n=40), log-rank test). **C** Comparison of DNA methylation patterns in normal and tumor tissues of patients with LUAD. **D** Correlation between DNA methylation patterns and H2AJ expression in patients with LUAD. Error bars show the mean ± standard error of the mean (SEM) of three biological replicates; **P* < 0.05, ****P* < 0.001 vs. control.

**Supplementary Fig. S2** *In vivo* analysis of metastasis. **A** Luminescence signals of A549 cells of all mice following intravenous injection. **B** Luminescence signals of A549 cells in lungs of all mice after sacrifice.

**Supplementary Fig. S3** Histological analysis of metastasis. **A** Metastatic nodules in lungs of all mice after sacrifice. **B** Hematoxylin and eosin (H&E) staining of all mouse lung tissues each groups. Scale bar = 200 μm.

**Supplementary Fig. S4** Validation of association with H2A.J and histone modification. **A** Profile plots of FLAG-H2A.J ChIP-Seq signal profiles at the endogenous H2A.J binding summit region in *H2AJ* overexpressing A549 cells. **B** Profile plots of endogenous H2A.J ChIP-Seq signal profiles at the FLAG-H2A.J binding summit region in *H2AJ* overexpressing A549 cells. **C** Distribution of the human genome (hg19). **D** Genomic distribution of endogenous H2A.J ChIP-Seq peaks in *H2AJ* overexpressing A549 cells. **E** Association of the endogenous H2A.J peaks in *H2AJ* overexpressing A549 cells with various histone markers region. **F** Association of H2A.J peaks with the public A549 chromHMM analysis data. The data are shown as the ratio of the H2A.J binding peak overlapping in the peak regions of the chromHMM 15 states. **G** ENCODE histone ChIP-Seq signal profiles in the *H2AJ* overexpressed endogenous H2A.J binding summit region. **H** Heatmaps and violin plots of endogenous H2A.J ChIP-Seq read distribution profiles and heatmaps of three gene groups classified by their expression levels in *H2AJ* overexpressing A549 cells.

**Supplementary Fig. S5** Integrated analysis of four putative target genes. Level of RNA-seq and Distributions of the Flag-H2A.J and H3K27me3 signals at the genomic loci of putative target genes in ectopic *H2AJ*-overexpressed A549 cells. The shaded boxes represent the promoter sites to which H2A.J (blue) and H3K27me3 (red) binds in the ChIP-seq analysis.

**Supplementary Fig. S6** Validation of H2A.J mechanism in A427 cell line **A** Binding to promoter regions of *TMEM158* and unchanged gene (*GAPDH*) using a ChIP-qPCR assay. **B** RT-qPCR and immunoblot validation of TMEM158 in *H2AJ* overexpressing A427 cells. **C** H3K27me3 at *TMEM158* promoter in *H2AJ* overexpressing A427 cells. **D** Results of Invasion assays with *H2AJ*, *TMEM158* overexpression alone or in combination. **E** Immunoblot validation of Flag-H2A.J after ectopic *Flag-H2AJ* with or without *TMEM158* overexpression in A549 cells. **F** The immunoprecipitation analysis for interaction between H2A.J and EZH2 after ectopic *H2AJ* overexpression in A427 cells. **G** Binding of EZH2 at *TMEM158* promoter in *H2AJ* overexpression condition in A427 cells. **H-I** H3K27me3 at *TMEM158* promoter (H) and RT-qPCR validation of *TMEM158* expression (I) in *H2AJ* overexpressing A427 cells alone or with *EZH2* knockdown. **J** Repression of transcriptional machinery on *TMEM158* promoter by *H2AJ* overexpression in A427 cells. Error bars show the mean ± standard error of the mean (SEM) of 3 biological replicates, **P* < 0.05, ***P* < 0.01, ****P* < 0.001 vs control; n.s; not significant

**Supplementary Fig. S7** SQ motif of H2A.J is involved in the invasiveness of LUAD through the regulation of *TMEM158* expression. **A** Sequence alignment of canonical H2A subtypes (H2A1, H2A2) and H2A.J. H2A.J is distinguished by A11V and the presence of an SQ motif near the C-terminus (red box). **B** Binding of EZH2, and H3K27me3 on *TMEM158* promoter after *H2AJ* wildtype or mutant overexpression. **C-D** TMEM158 mRNA (C) and protein expression (D) after *H2AJ* wildtype or mutant overexpression in A549 cells. **E** Luciferase assay results using the reporter system with *TMEM158* promoter following *H2AJ* wildtype or mutant overexpression in HEK293 cells. **F-G** Results of invasion (F) and proliferation assays (G) after *H2AJ* wildtype or mutant overexpression in A549 cells. Scale bar = 200 μm. Error bars show the mean ± standard error of the mean (SEM) of 3 biological replicates, **P* < 0.05, ****P* < 0.001 vs control.

**Supplementary Fig. S8.** Validation of H2AJ expression and phenotypes in A427 cell line and another LUAD dataset. **A** Comparison of *TMEM158* expression in normal and tumor tissues of patients with LUAD. **B** Overall survival rates of patients with LUAD classified by *H2AJ* expression (*P* = 5.9 × 10^-2^ for GSE19188 (n=40), log-rank test). **B-C** Results of immunoblot validation, invasion, and proliferation assay after ectopic *TMEM158* overexpression (B) and knockdown (C) in A427 cells. Upper band is Flag-tagged TMEM158 proteins and lower band is endogenous proteins (black triangle). Scale bar = 200 μm. **D** Correlation of *H2AJ* and *TMEM158* expression (Spearman’s rank correlation coefficient). **E** Overall survival of LUAD patient groups stratified by *H2AJ* and *TMEM158*. The number of patients (n) in each subgroup (*H2AJ*-high/*TMEM158*-low, *H2AJ*-low/*TMEM158*-low, *H2AJ*-high/*TMEM158*-high, and *H2AJ*-low/*TMEM158*-high) is indicated for the GSE19188 (n = 16, 1, 19, 4). Error bars show the mean ± standard error of the mean (SEM) of 3 biological replicates, **P* < 0.05, ****P* < 0.001 vs control.
